# Metagenomic psychrohalophilic xylanase from camel rumen investigated for bioethanol production from wheat bran using *Bacillus subtilis* AP

**DOI:** 10.1038/s41598-022-11412-4

**Published:** 2022-05-17

**Authors:** Marzieh Rajabi, Farahdokht Nourisanami, Kamran Khalili Ghadikolaei, Mohammad Changizian, Kambiz Akbari Noghabi, Hossein Shahbani Zahiri

**Affiliations:** Department of Energy and Environmental Biotechnology, National Institute of Genetic Engineering and Biotechnology (NIGEB), Tehran, Iran

**Keywords:** Biotechnology, Industrial microbiology

## Abstract

Bioethanol produced from lignocellulosic biomass is regarded as a clean and sustainable energy source. The recalcitrant structure of lignocellulose is a major drawback to affordable bioethanol production from plant biomass. In this study, a novel endo-1,4-xylanase, named Xyn-2, from the camel rumen metagenome, was characterized and evaluated for hydrolysis of agricultural wastes. The enzyme was identified as a psychrohalophilic xylanase with maximum activity at 20 °C, keeping 58% of the activity at 0 °C, and exhibiting twice as much activity in 0.5–4 M NaCl concentrations. Xyn-2 was able to hydrolyze wheat bran (100%), sunflower-seed shell (70%), wheat straw (56%), rice straw (56%), and rice bran (41%), in the relative order of efficiency. Besides, the ethanologenic *B. subtilis* AP was evaluated without and with Xyn-2 for bioethanol production from wheat bran. The strain was able to produce 5.5 g/L ethanol with a yield of 22.6% in consolidated bioprocessing (CBP). The contribution of Xyn-2 to ethanol production of *B. subtilis* AP was studied in an SSF system (simultaneous saccharification and fermentation) giving rise to a significant increase in ethanol production (p ≤ 0.001) to a final concentration of 7.3 g/L with a yield of 26.8%. The results revealed that the camel rumen metagenome might be an invaluable source of novel xylanolytic enzymes with potential application in lignocellulosic biomass valorization. At the same time, the results suggest that *B. subtilis* with a diverse carbon-source preference and sophisticated systems for production and secretion of enzymes might be a promising candidate for strain development for bioethanol production from plant biomass. It might be assumed that the fortification of *B. subtilis* enzymatic arsenal with select xylanolytic enzymes from camel rumen metagenome may have a great impact on bioethanol production.

## Introduction

The environmental issues arising from excessive consumption of fossil fuels have raised attention to alternative renewable biofuels. Biofuels can be produced from plant biomass in a short time, in contrast to fossil fuel which requires millions of years for formation. Agricultural and forestry wastes such as straws, husks, brans, corn stovers, and woods are regarded as the most promising feedstocks for biofuel production, because they are abundant, renewable, and, importantly, not used for human food^1^. In recent decades, many countries have developed initiatives for bioethanol production using plant biomass wastes^2,3^. This strategy would be beneficial in waste recycling to produce bioethanol as a clean energy and dealing with the consequences of excessive fossil-fuel consumption during the last years^4–6^.

Plant biomass is essentially made up of lignocellulose which is a complex substance composed of carbohydrate (cellulose and xylan) and phenolic (lignin) polymers. The components of lignocellulose are entangled together by covalent and non-covalent interactions into a tough structure that gives strength to plant cell wall^7^. Xylanolytic enzymes are of great importance in the decomposition of plant cell wall and, therefore, plant biomass utilization in many applications^8^. Xylanolytic enzymes not only are directly involved in the degradation of plant biomass but also exhibit synergistic effects on cellulolytic enzymes by increasing substrate availability through disentanglement of the cellulose-hemicellulose complex structure of biomass. Xylanases are classified based on primary structure comparisons mainly in families 5, 7, 8, 10, 11, and 43 of glycoside hydrolases according to CAZy (carbohydrate-active enzyme) database^9^. Family 5 and 10 contain (β/α)_8_ structure, family 7 and 11 have β-Jelly roll structure, family 8 shows (α/α)_6_ structure, and family 43 exhibits 5 blade β-propeller structure^10^. The hydrolysis of xylan to xylose monomers requires at least two enzymes including *endo*-1,4-β-d-xylanase (EC 3.2.1.8) and β-d-xylosidase (E.C.3.2.1.37). The endo‐1,4‐β‐xylanase exhibits a more significant effect on hemicellulose degradation as the enzyme breaks xylan chain from inside providing more reducing ends for the action of β-d-xylosidase^7^. In addition, endo‐1,4‐β-D‐xylanase is more important in economic terms due to the high demands and a growing market for the enzyme in food and biofuel industries^11,12^. Various endo-xylanases have been obtained from cultured microorganisms. Recently, the research for novel enzymes has been extended to uncultured microorganisms by the advent of metagenomics. Thereby, many novel xylanases with unprecedented characteristics have been discovered using metagenomes of goat rumen, cow rumen, camel rumen, compost, hot spring sediments, and termite gut^13–18^.

Psychrophilic enzymes exhibit high catalytic activity at moderate and cold temperatures. The enzymes have flexible structures to counteract the rigidity that is induced by cold temperatures. As a result, they exhibit higher catalytic activity at cold at the expense of a lowered melting temperature and thermal stability as compared to mesophilic and thermophilic enzymes. The use of psychrophilic enzymes can be beneficial to improve efficiency. In addition, they can also help to reduce costs and environmental impacts arising from excessive energy consumption. Therefore, psychrophilic enzymes show great potential for application in food, bioremediation, waste recycling, detergent, and pharmaceutical industries^19,20^.

The feedstock availability is one important consideration for affordable bioethanol production. In this regard, various biomass wastes have been investigated for feedstock based on geographical availability. Wheat bran, accounting for 14–19% of the grain, is an abundant by-product of the wheat milling industry. Only a small part of wheat bran is currently utilized for dietary food supplements and animal feed. Therefore, wheat bran is potentially an available feedstock for bioethanol production in wheat-growing countries like Iran with an estimated wheat production of about 13 Mt per year. Wheat bran consists of starch (10–20%), non-starch polysaccharides (46%), proteins (15–22%), and lignin (4–8%)^21^. Glucuronoarabinoxylans are the main constituents (about 70%) of the non-starch polysaccharides. Thus, xylanases are important enzymes involved in the saccharification of wheat bran. In the present study, a novel endo-xylanase (Xyn-2) from the camel rumen metagenome was characterized and evaluated for saccharification of wheat bran. The coding gene was cloned and expressed in *Escherichia coli* and the recombinant xylanase was purified for biochemical characterization. In addition, the crude enzyme was used for saccharification of wheat bran and ethanol production. For ethanol production, an engineered ethanologenic *Bacillus subtilis* strain was deployed. The efficiency of the strain for bioethanol production from wheat bran and the synergistic effect of Xyn-2 were investigated.

## Materials and methods

### Materials, culture mediums, and strains

Tryptone and yeast extract were purchased from Merck (Darmstadt, Germany). DNA polymerase, T4 DNA ligase, restriction enzymes, and DNA marker were purchased from Thermo Fisher Scientific (Waltham, USA). Protein marker was obtained from SinaClon (Tehran-Iran). The pET-26b(+) vector and the strains DH5α and BL21(DE3) of *Escherichia coli* were from Novagen (Madison, USA). The DH5α and BL21(DE3) strains were used for gene cloning and expression, respectively.

*E. coli* DH5α is not capable of homologous recombination due to a *recA* mutation that ensures higher stability of insert genes. In addition, a mutation in *endA1* decreases endonuclease degradation of plasmids resulting in higher plasmid transfer rates during transformation. In contrast, *E. coli* BL21 was engineered for protein expression through the deletion of some proteases. As a result, the recombinant proteins remain secure from proteolytic digestion. Also, the strain carries the gene encoding T7 RNA polymerase by which plasmids containing strong T7 promoter can be used for high levels of protein production. The strains were cultured on Luria–Bertani (LB) medium containing 1% tryptone, 0.5% yeast extract, and 1% NaCl at 37 °C. The transformation of *E. coli* strains was performed by the heat shock method and the transformants were selected on LB agar plates with kanamycin (50 µg/mL) according to standard protocols^22^. The PCR purification and plasmid extraction kits were purchased from GeneAll (Seoul, Korea). The Ni–NTA protein purification resin was purchased from Qiagen (Hilden, Germany). The ethanologenic *Bacillus subtilis* AP [WB600, *ldh*::pDH*tldh*, pHY*adh*S:*pdc*Z] was available from a previous study^23^. This stain was deficient in lactate fermentation due to an insertion mutation in the lactate dehydrogenase gene (*ldh*). Instead, it was able to produce ethanol by a synthetic pathway composed of *S. cerevisiae* alcohol dehydrogenase (*adhS*) and *Z. mobilis* pyruvate decarboxylase (*pdc*Z). The related genes were expressed in *B. subtilis* AP in the form of a chimeric gene (*adh*S:*pdc*Z) using pHY300PLK as an expression vector.

### Metagenome sequencing and gene finding

The camel rumen metagenome was obtained by a previous study^24^. The metagenome was sequenced at the Beijing Genome Institute (BGI, Shenzhen, China). For this purpose, a library was prepared using a Nextera DNA Library Preparation Kit, according to the manufacturer’s protocol (Illumina, San Diego, CA, USA). For this purpose, 200 µg of the metagenomic DNA was sheared using Covaris sonicator (Covaris Inc, Massachusetts, USA), and the resulting fragments were end-repaired, adenylated, and ligated with Illumina sequencing adaptors. DNA fragments of about 350 nt were purified using the Agencourt AMPure XP beads (Beckman Coulter, Beverly, MA, USA). After PCR amplification, the library was sequenced in paired-end mode on an Illumina HiSeq 2000 Sequencing System. High-quality reads were assembled into contigs using IDBA-UD v1.1 (http://www.cs.hku.hk/∼alse/idba_ud) and SPAdes v3.10 (https://cab.spbu.ru/software/spades/) software^25,26^. The paired-end reads were mapped to scaffolds using BBMap v36.92 (https://jgi.doe.gov/data-and-tools/bbtools/bb-tools-user-guide/installation-guide/) to determine the percentage of assembled reads and the coverage profiles of scaffolds. The assembled scaffolds of more than 200 nt were retained and subsequently submitted to the Integrated Microbial Genomes (IMG) with submission ID 142919 and project number Ga0206072. The open reading frames (ORFs) were identified using Prodigal v2.6.3^27^. The translated amino acid sequences of the ORFs were annotated against Clusters of Orthologous Genes (COGs)^28^, Protein families database (Pfam)^29^, SEED subsystem^30^, and the KEGG pathways database^31^ using COGNIZER v0.9b (http://metagenomics.atc.tcs.com/cognizer)^32^.

### Sequence analysis and heterologous expression of *xyn-2*

An ORF annotated as coding for a xylanase, tentatively named *xyn-2*, was selected for in silico and functional analysis. The similarity analysis of *xyn-2* was performed by blastn and blastx suites (version 2.12.0) at NCBI (https://blast.ncbi.nlm.nih.gov/Blast.cgi) and EMBL-EBI (https://www.ebi.ac.uk/Tools/sss/ncbiblast/). Blastn was conducted using default parameters with a standard database (nucleotide collection) and blastx was performed using standard genetic code and non-redundant protein sequence database. The domain prediction and classification of Xyn-2 were performed by Pfam (http://pfam.xfam.org) and InterPro (https://www.ebi.ac.uk/interpro/). The physicochemical properties of Xyn-2 were analyzed by ProtParam tool (http://web.expasy.org/protparam/). The tertiary structure of Xyn-2 was modeled by Phyre2 server (http://www.sbg.bio.ic.ac.uk/~phyre2) and visualized by PyMOL (https://pymol.org/2/). The sequence analysis, primer design, and *in-silico* cloning were conducted using Vector NTI advance 10 software (http://www.invitrogen.com). The gene was amplified by PCR (polymerase chain reaction) using the oligonucleotides 5′-AGA AGC TTA AAC AAA GTT TTA AAA AGG CTC-3′ and 5′-ATC TCG AGT TTC TTG AAC AAT AAT GGA G-3′ as former and reverse primers, respectively. The camel rumen metagenome which was available from our previous study was used for template^24^. The PCR amplification was conducted using *Pfu* DNA polymerase under the following conditions: 95 °C for 5 min, 30 cycles of 95 °C for 30 s, 51 °C for 30 s, 72 °C for 3.5 min, followed by 72 °C for 10 min. The PCR product was restricted by *Hin*dIII and *Xho*I, purified, and ligated with linearized pET-26b(+), previously restricted by the same enzymes. Thereby, *xyn-2* was inserted in pET-26b(+) in frame with the *pel*B sequence at upstream and the His-tag sequence at its downstream (Supplementary Fig. S1). The resulting recombinant plasmid, pETxyn-2, was used for the transformation of *E. coli* DH5α, and transformants were selected on LB medium with kanamycin (50 µg/mL). The recombinant plasmid was confirmed by sequencing to make sure of the authenticity of the cloning procedure. pETxyn-2 was then transformed in *E. coli* BL21(DE3) for gene expression. The resulting recombinant *E. coli* was cultured in LB/kanamycin medium at 37 °C, 200 rpm. When the optical density of the culture medium measured about 0.8 (600 nm), the gene expression was induced by 0.3 mM IPTG (Isopropyl β-d-1-thiogalactopyranoside) for 24 h at 25 °C, 120 rpm. The recombinant xylanase was obtained from periplasmic space. For this purpose, cells were isolated by centrifugation (4000*g*, 15 min) and resuspended in Tris–HCl (30 mM, pH 8) containing 1 mM EDTA and 20% sucrose. This suspension was kept on ice for 10 min with gentle shakes at 2 min intervals and then centrifuged (8000*g*, 4 °C, 20 min). The resulting pellet was resuspended in 5 mM MgSO_4_ and kept on ice for 10 min with gentle shakes. After centrifugation (8000*g*, 4 °C, 20 min) the supernatant was separated and dialyzed overnight against the dialysis buffer (50 mM Na_2_HPO_4_, 300 mM NaCl, 10 mM imidazole, pH 8). The recombinant xylanase was purified by Ni–NTA (nickel nitrilotriacetic acid) resin using standard protocols recommended by the manufacturer (Qiagen). The purified enzyme was analyzed by SDS-PAGE (sodium dodecyl sulfate–polyacrylamide gel electrophoresis) and its concentration was determined by the Bradford method. For the crude enzyme preparation, the cells expressing Xyn-2 were harvested from a 50 mL culture medium by centrifugation (10,000*g*, 10 min). The cells were resuspended in 10 mL lysis buffer (50 mM Na_2_HPO_4_, 300 mM NaCl, 10 mM Imidazole, 1 mg/mL lysozyme, pH 8), hold on ice for 30 min, and then disrupted by sonication. Finally, the cell debris was separated by centrifugation (10000*g*, 10 min, 4 °C) and the resulting supernatant was used as the crude enzyme for substrate hydrolysis. The cell lysate of *E. coli* BL21 harboring pET-26b(+) was used as a control in the experiments conducted by the crude enzyme to exclude any background activity that might be presumed in *E. coli* BL21. The sequences of *xyn-2* and pETxyn-2 were submitted to GenBank with the accession numbers KX644148.1 and OL449269, respectively.

### Assay of xylanase activity

The xylanase activity was assayed by measuring the amount of reducing sugars released from xylan as a result of the enzyme function (Miller 1959). The assays were conducted in microtubes with 90 μl of citrate buffer (pH 5) containing 1 mg/mL oat-spelt xylan and 10 μl of purified enzyme (1.63 mg/mL). The reactions were stopped after 10 min incubation at an appropriate temperature by adding three volumes of DNS (3,5-dinitrosalicylic acid) and heating in boiling water for 10 min. The light absorbance of the developing color was measured at 540 nm to calculate the concentration of reducing sugars using a standard curve. For the control reaction, the enzyme was excluded and instead 10 μl of citrate buffer (pH 5) was added. The control reaction was used as a blank to calibrate the spectrophotometer. The standard curve was prepared using xylose at 0.1, 0.2, 0.3, 0.4, 0.5, 0.6, and 0.8 mg/mL concentrations. Each xylose concentration (100 µl) was treated with DNS as described before, and the absorbance of the developing color was read at 540 nm. The data were fit into a line equation using the linear regression by SigmaPlot 14.0. The line equation was used to calculate the concentration of reducing sugars released from xylan as a result of Xyn-2 activity. One unit of enzyme activity was defined as the amount of enzyme required to produce one micromole of reducing sugars per minute. The xylanase activity on some crop wastes including wheat bran, wheat straw, rice bran, rice straw, and sunflower-seed shells was analyzed using the unpurified enzyme. The crop wastes, prepared from a local market, were washed in distilled water, dried at 50 °C, and milled to homogenous particles of about 0.1 mm. To assay the enzyme activity, 1 mg of each biomass was treated with 100 μl (0.3 U) of the crude enzyme for one hour at 20 °C. The controls were the same as the tests except that the substrates were treated with 100 μl of the buffer instead of crude enzyme. Then the concentration of reducing sugars released as a result of the enzyme activity was measured by the DNS method as described before. For pretreatment, wheat bran was washed in water and dried at 50 °C to a constant weight. The dried biomass (3 g) was added to 17 mL of either distilled water, artificial seawater, 0.3% HCl, 0.3% HCl in artificial seawater, 0.1% NaOH, or 0.1% NaOH in artificial seawater and was autoclaved at 120 °C for 20 min. The biomass was then separated by filtration using Whatman paper No.1 and dried at 50 °C to a constant weight. The dried preparations of wheat bran were each used as a substrate for xylanase assay to determine the impact of the different pretreatments on the substrate digestibility. The artificial seawater used in this study was composed of 26.29 g/L NaCl, 0.74 g/L KCl, 0.99 g/L CaCl_2_, 6.09 g/L MgCl_2_·6H_2_O, and 3.94 g/L MgSO_4_·7H_2_O.

### SDS-PAGE and zymography

Protein electrophoresis (SDS-PAGE) was conducted according to the Laemmli method using a 12% polyacrylamide gel (Laemmli 1970). The native-PAGE for zymography was performed likewise with the exception that SDS and β-mercaptoethanol were eliminated from the protocols of the running buffer, polyacrylamide gel, and loading buffer preparation. In addition, 1% oat-spelt xylan was included in the native polyacrylamide gel and the electrophoresis was conducted at 4 °C. In the end, the gel was washed with distilled water and incubated in citrate buffer (pH 5) at 37 °C for 1 h. The native gel was then stained in 0.1% Congo Red solution for 30 min and finally was developed in 1 M NaCl solution.

### Effects of pH, temperature, NaCl, ions, inhibitors, and denaturants on enzyme activity

The effects of physicochemical conditions on the enzyme activity were analyzed at various pH and temperatures as well as in the presence of chemicals including ions, inhibitors, and denaturants that are commonly used in enzyme studies.

Various buffers including citrate buffer (pH 4–6), phosphate buffer (pH 6–8), and glycine–NaOH buffer (pH 8–10) were used to analyze the relative enzyme activity under various pH conditions at 35 °C. The highest enzyme activity was taken as 100%, and the activities obtained at other pH conditions were expressed as a percentage of that. The effect of temperature in the range of 0–50 °C was studied on the enzyme activity in citrate buffer at pH 5. In other experiments, the enzyme activity was assayed at pH 5 and 20 °C as optimum conditions. The influence of NaCl at 1–5 M and urea in the range of 0.2–1.7 M concentrations were studied by standard assays conducted at the optimum pH and temperature obtained as mentioned above. The impacts of detergents including 10% and 20% of Triton, Tween 20, and Tween 80; 0.1% SDS (sodium dodecyl sulfate); 1% and 3% of 2-ME (2-mercaptoethanol) and GuHCl (guanidium hydrochloride); 0.1% ammonium persulphate; 1 mM and 5 mM of EDTA (ethylenediaminetetraacetic acid) and PMSF (phenylmethylsulfonyl fluoride); and 10% DMF (dimethylformamide) were studied. Ions including Zn^2+^, Cu^2+^, NH_4_^+^, Mg^2+^, Mn^2+^, Fe^2+^, Co^2+^, Ca^2+^, Cd^2+^,Ba^2+^, Na^+^, and K^+^ were studied at 5 mM and 10 mM concentrations. Solvents including dichloromethane, methanol, ethanol, isobutanol, acetone, hexane, toluene, chloroform, benzene, xylene, and cyclohexane were all studied at 10% and 20% final concentrations. The control was a standard enzyme reaction, as described before, without any additional substance. The activity obtained from the control was taken as 100% and those obtained under the influence of ions, inhibitors, and denaturants were expressed relatively. The relative activities were calculated by the following equation:

$$ Relative\,activity \left( \% \right) = \frac{activity\,of\,sample }{{actiivty\,of\,controle}} \times 100. $$

The data presented in the manuscript are the mean ± standard deviation of three independent experiments. The statistical analyses and presentation of data were conducted by SigmaPlot 14.0 (www.sigmaplot.com). The one-way ANOVA followed by Tukey’s test was used to determine the significance of differences in enzyme activities (Supplementary file).

### Bioethanol production

*B. subtilis* AP was used for bioethanol production from wheat bran. The strain was available from a previous study and just renamed from NS:Z to AP for simplicity^23^. The AP stands for alcohol dehydrogenase and pyruvate decarboxylase. The strain AP carried a chimeric gene constructed from *S. cerevisiae adhI* (alcohol dehydrogenase coding gene) and *Z. mobilis pdc* (pyruvate decarboxylase coding gene). The chimeric gene was expressed under the control of *Tet* promoter of pHY300PLK conferring ethanologenic phenotype to the harboring strain AP. For ethanol production, wheat bran pretreated with HCl and artificial seawater was added (10% w/v) to 2YT medium without and with Xyn-2 (13.5 U). The resulting mediums, named PB (2YT plus Pretreated Bran) and PBX (2YT plus Pretreated Bran and Xyn-2), along with UB medium (2YT plus Unpretreated Bran) as control were used for bioethanol production by *B. subtilis* AP. All cultures for bioethanol production were conducted in 1 L flasks containing 100 mL of each medium that were inoculated with *B. subtilis* AP to an initial optical density of 0.1 (600 nm) and incubated at 37 °C, 180 rpm for 98 h. Samples were taken after 48 h and 98 h of fermentation to measure the bioethanol content. The residual solids were collected by centrifugation and dried at 50 °C to measure the weight of the remaining biomass. The one-way ANOVA followed by Tukey’s test was used to determine the significance of differences in bioethanol production and biomass utilization (Supplementary file).

### Ethanol measurement

A Varian CP-3800 gas chromatograph equipped with a CP-Wax 57

CB column, a 1041 injector, and a flame ionization detector (FID) were used to measure ethanol concentration. The helium of high purity was used as the carrier gas at a flow rate of 1 mL/min. The injection port temperature was set at 200 °C, and the detector temperature was at 250 °C.

For determination of ethanol concentration in samples, a series of known ethanol concentrations (0–10 g/L) supplemented with 2 g/L methanol as internal standard was prepared in distilled water and injected (1 µL) in the GC equipment. A scatter plot was made using the proportion of ethanol/methanol peak areas versus the known ethanol concentrations. The scatter plot was fitted into a line plot by linear regression and the ethanol concentration of unknown samples was calculated by the resulting line equation. The ethanol yield (%) was calculated using the following equation:

$Y = \frac{\left[ E \right]}{{\left[ S \right] \times F \times 1.111 \times 0.51}} \times 100$, where [E] is the ethanol concentration (g/L), [s] is the solubilized substrate (g/L), F is the polysaccharide fraction, 1.111 and 0.51 are coefficients to reflect polysaccharide hydrolysis and the theoretical maximum of ethanol production, respectively.

This study was in compliance with relevant institutional, national, and international guidelines and legislation.

## Results

### Identity analysis and heterologous expression of *xyn-2* in *E. coli*

**Figure 1:**
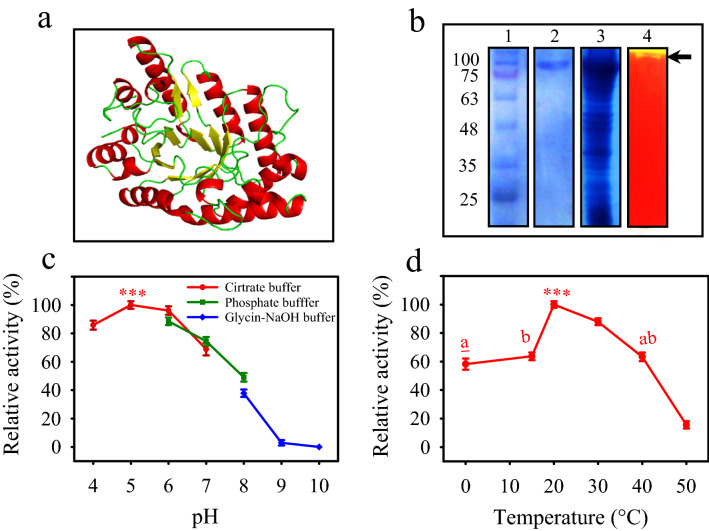
(**a**) Modeled structure of Xyn-2. The (β/α)_8_ barrel is a characteristic feature of family 10 xylanases (**b**) SDS-PAGE and native PAGE of purified Xyn-2. Lane 1—molecular weight marker, lane 2—purified Xyn-2, lane 3—cell lysate of *E. coli* BL21(DE3) expressing Xyn-2, lane 4—native PAGE and activity staining of Xyn-2 with Congo-red (from supplementary Fig. S3). The arrow indicates the yellowish band of xylan hydrolysis (**c**) Effect of pH on the activity of Xyn-2 measured by running reactions in various pH buffers at 35 °C. The highest enzyme activity, obtained at pH 5, was taken as 100%, and the enzyme activities obtained at other pH conditions were expressed as a percentage of the highest activity (**d**) Influence of temperature on the activity of Xyn-2 measured at pH 5 in citrate buffer. The highest enzyme activity, obtained at 20 °C, was taken as 100%, and the enzyme activities obtained at other temperatures were expressed as a percentage of the highest activity. The significant difference between the peak value and other values of enzyme activity is indicated by the stars (p ≤ 0.001). Letters reflect values that are not significantly different from each other.

The sequencing and gene-finding analyses of the camel rumen metagenome resulted in the identification of *xyn-2* as coding for an endoxylanase*.* The nucleotide blast of *xyn-2* (GenBank: KX644148.1) retrieved only one identical gene (GenBank: FJ713437.1) belonging to *Prevotella ruminicola* with just 21% coverage*.* However, the translated amino acid sequence of FJ713437.1 showed 62% identity with that of *xyn-2* (Supplementary Fig. S2). The protein blast of *xyn-2* using blastx retrieved hypothetical xylanase sequences reported from genome and metagenome annotations. The analyses conducted using Pfam and InterPro predicted a glycoside hydrolase family 10 domain in Xyl-2 structure. In addition, Xyn-2 modeled tertiary structure revealed a (β/α)_8_ catalytic domain that is a characteristic feature of family 10 xylanases (Fig. 1a). The PDB molecule used in the homology modeling belonged to a psychrophilic glycoside hydrolase family 10 endo-β-1,4-xylanase. This molecule (PDB code: c5d4yA) with the highest identity (69%) to Xyl-2 was identified as the best template among other confident templates. According to the observations, *xyn-2* was provisionally identified as coding for a family 10 endo-1,4-beta-xylanase composed of 729 amino acids, with a molecular mass of 83 kD and a *pI* of 8.46.

The gene was successfully expressed in *E. coli* BL21(DE3) and the resulting protein was purified using the Ni–NTA resin from the periplasmic fraction. The purity of the enzyme was confirmed by SDS-PAGE showing a single band corresponding to the theoretical mass of the recombinant Xyn-2 (88 kD). The native-PAGE containing 1% OSX revealed that the purified enzyme was a functional xylanase with the ability to in-gel digestion of OSX leaving a yellowish area in the red background after staining with Congo Red (Fig. 1b).

### Effects of physicochemical conditions on the enzyme activity

The effects of physicochemical conditions on the enzyme activity were analyzed at various pH and temperatures as well as in the presence of chemicals including inhibitors and denaturants that are commonly used in enzyme studies. The enzyme reactions conducted at various pH conditions revealed that Xyn-2 was an acidic xylanase with its best activity at pH 5. The enzyme was able to exhibit more than 85% of its highest activity in the range of pH 4–6. However, the enzyme activity was dropped by 25% and 50% at neutral pH 7 and the mild alkaline pH 8, respectively. Higher alkaline conditions resulted in the total loss of activity at pH 9 and pH 10 (Fig. 1c). The temperature profile of the xylanase activity showed that Xyn-2 was best active at 20 °C and retained more than 58% of the activity at lower temperatures down to 0 °C. In contrast, the enzyme was not tolerant of heat so that its activity decreased severely from 63 to 16% by the increase of temperature from 40 to 50 °C. The results indicate that Xyn-2 must be a psychrophilic enzyme with maximum activity at moderate temperatures (Fig. 1d).

**Figure 2:**
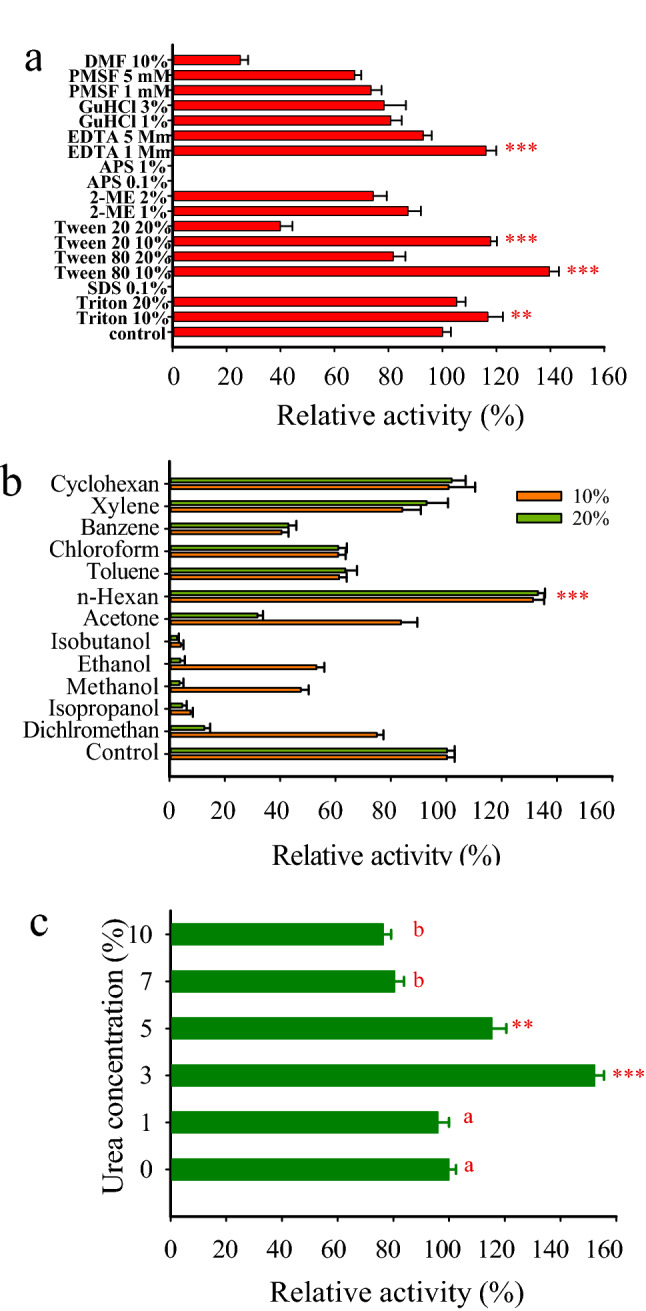
(**a**) Effects of surfactants, reducing and oxidizing agents, and inhibitors on Xyn-2 activity. (**b**) Effects of organic solvents on the enzyme activity. (**c**) Relative activity of Xyn-2 in various concentrations of urea. All reactions were conducted at 20 °C and pH 5 in citrate buffer. The enzyme activity in the buffer-only condition (control) was taken as 100% and the activities obtained under the influence of the inhibitors and denaturants were expressed relative to that. The stars indicate a significant increase in the enzyme activity at p ≤ 0.001 (***) and p ≤ 0.01 (**) compared to control. The groups indicated by the same letter are not significantly different.

Xyn-2 showed to be quite tolerant of non-ionic surfactants so that it could tolerate high concentrations (10% and 20%) of Triton X100, Tween 80, and Tween 20 while the SDS as an ionic detergent was able to deactivate the enzyme even at a low concentration (0.1%). The reducing agent 2-ME at 1% and 2% concentrations decreased the enzyme activity of Xyn-2 only by 13% and 26%, respectively, but the enzyme was thoroughly deactivated by the oxidizing agent APS at a much lower concentration (0.1%). The enzyme activity was not much affected by EDTA indicating the Xyn-2 must not be a metal enzyme and its activity is not dependent on ions. Also, the enzyme was not severely influenced by GuHCl and PMSF (Fig. 2a).

**Figure 3:**
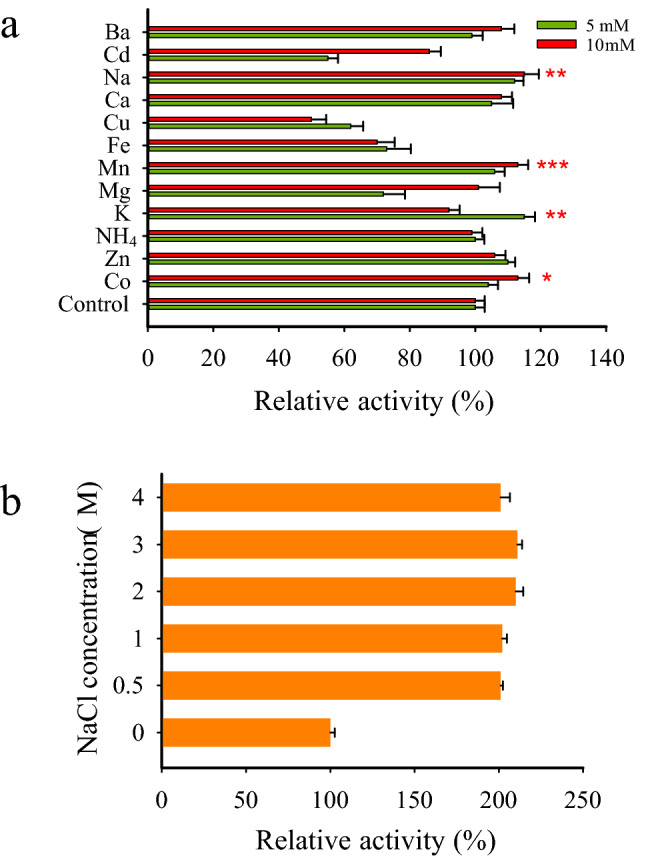
(**a**) Relative activity of Xyn-2 in the presence of various cations at 5 mM and 10 mM concentrations. The amount of enzyme activity in the absence of the cations (control) was taken as 100% and the activity in the presence of each cation was expressed relative to that. (**b**) Influence of NaCl at high concentrations on Xyn-2 activity. The amount of enzyme activity in the absence of NaCl (control) was taken as 100% and the activities obtained in various concentrations of NaCl were expressed as a percentage to that. All reactions were performed at 20 °C, in citrate buffer (pH 5). The stars indicate a significant increase in the enzyme activity at p ≤ 0.001 (***), p ≤ 0.01 (**), and p ≤ 0.05 (*) compared to the control.

The evaluation of the impact of various solvents on Xyn-2 showed that the enzyme, in general, was more tolerant of immiscible solvents than the miscible ones. Hexane was the only solvent that improved the enzyme activity at both 10% and 20% concentrations. The enzyme was tolerant of cyclohexane with no detected loss of activity at both tested concentrations. Other immiscible solvents including xylene, toluene, chloroform, and benzene reduced the enzyme activity in the range of 16–60%. In contrast, all miscible solvents showed adverse effects on the enzyme activity in the following order of magnitude: Isobutanol > isopropanol > methanol > ethanol > dichloromethane > acetone. The effects of isobutanol and isopropanol were more severe than other solvents decreasing the enzyme activity to 4% and 7.6% at 10% concentration, respectively (Fig. 2b). The enzyme was not only highly tolerant of urea in the range of 0.2–1.7 M concentrations but also its activity was significantly improved by 52% in the presence of 0.5 M urea (Fig. 2c). The addition of various ions at 5 and 10 mM to the reaction mixture indicated that Xyn-2 was tolerant of severe inhibitory effects that might be imposed by the ions. However, some ions including Cu^2+^, Cd^2+^, Mg^2+^, and Fe^2+^ were different from others in that they partially reduced the enzyme activity (Fig. 3a). Interestingly, the activity of Xyn-2 was enhanced more than two times by NaCl at various concentrations in the range of 0.5–4 M. it may be assumed from this observation that Xyn-2 is a halophilic xylanase with a remarkable enhancement of activity in the presence of high concentrations of NaCl (Fig. 3b).

### Xyn-2 activity on natural substrates

**Figure 4:**
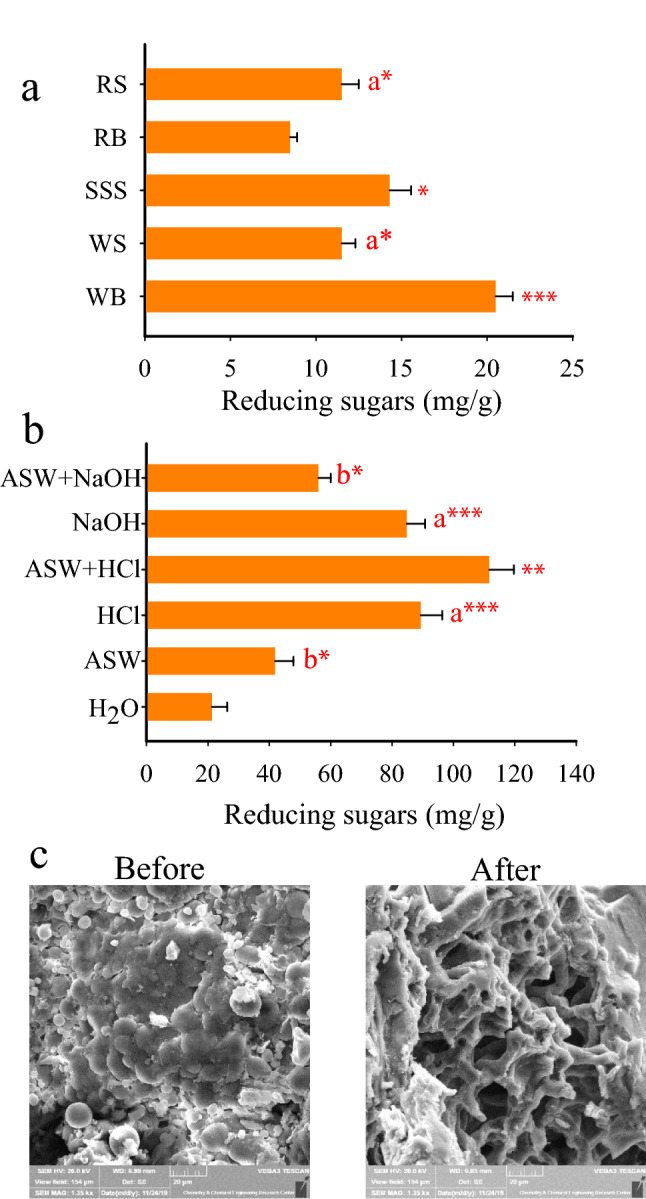
(**a**) Activity of Xyn-2 on wheat bran (WB), wheat straw (WS), sunflower-seed shell (SSS), rice bran (RB), and rice straw (RS). One mg of each biomass was treated with 100 μl (1.35 U) of crude enzyme for one hour at 20 °C. The activity of Xyn-2 on the substrates was analyzed by the measurement of reducing sugars. (**b**) Effects of various pretreatments including artificial seawater (ASW), hydrochloric acid (0.3% HCl), artificial seawater plus HCl (ASW + 0.3% HCl), sodium hydroxide (0.1% NaOH), and artificial seawater plus NaOH (ASW + 0.1% NaOH) on digestibility of wheat bran by Xyn-2. The effects were analyzed by the measurement of reducing sugars released as a result of the enzyme activity. (**c**) Electron micrographs of wheat bran pretreated with artificial seawater plus HCl before and after Xyn-2 hydrolysis. The significance of the difference in sugar concentration between nearby values is indicated by stars at p ≤ 0.001 (***), p ≤ 0.01 (**), and p ≤ 0.05 (*). The letters (a and b) indicate that there is no significant difference between means of the same letter.

As an important part of Xyn-2 characterization, some agricultural wastes including wheat bran, wheat straw, rice bran, rice straw, and sunflower seed shells, which are leftovers of widely cultivated crops in Iran, were used as substrates. These crop wastes are different from each other in relative amounts of cellulose, hemicellulose, and lignin as well as the complexity of the composite structure of cell walls. The analysis of Xyn-2 activity showed that the enzyme was able to hydrolyze physiochemically untreated wheat bran, sunflower-seed shell, wheat straw, rice straw, and rice bran in the order of magnitude. The enzyme activity on wheat bran was 2.4 times higher than rice bran, 1.8 times higher than wheat and rice straws, and 1.4 times higher than the sunflower-seed shell (Fig. 4a). Given that the highest activity of Xyn-2 was obtained with wheat bran, the substrate was used to analyze the effect of physicochemical pretreatments on its digestibility. The results indicated that the pretreatments could significantly improve the digestibility of wheat bran by Xyn-2. As a result, the enzyme activity on wheat bran was increased by 5.3, 4.2, 4, 2.6, and 2 times with 0.3% HCl in artificial seawater, 0.3% HCl, 0.1% NaOH, 0.1% NaOH in artificial seawater, and just artificial seawater compared to distilled water as control, respectively (Fig. 4b). The analysis of wheat bran by electron microscopy before and after treatment with Xyn-2 indicated that the enzyme can effectively degrade the plant biomass. As shown in Fig. 4c, the decomposition of wheat bran by the enzyme activity can be seen as the holes and frameworks that are left over after the removal of digestible contents.

### Bioethanol production

**Figure 5:**
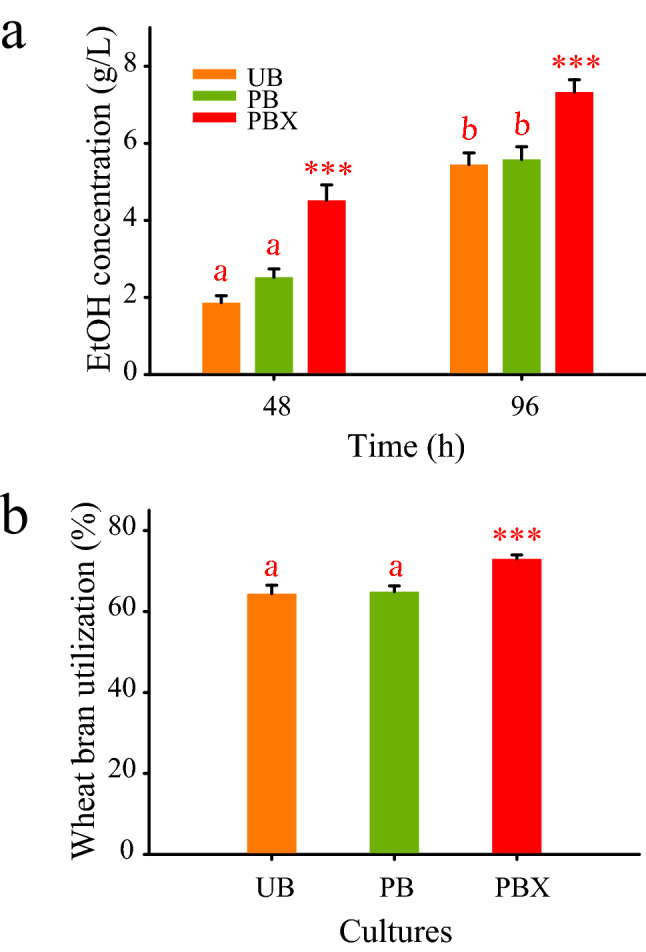
(**a**) Bioethanol production by *B. subtilis* AP using untreated wheat bran (UB), pretreated wheat bran (PB), and pretreated wheat bran plus Xyn-2 (PBX ) in 2YT medium. Pretreatment was conducted with artificial seawater plus 0.3% HCl. Xyn-2 was included in the PBX culture medium at 13.5 U/mL. (**b**) Wheat bran dissolution efficiency in each culture medium was estimated from the residual biomass at the end of fermentation. The significantly different mean values are indicated by stars (p ≤ 0.001). Letters reflect values that are not significantly different from each other.

Given that the highest activity of Xyn-2 was obtained on wheat bran, this substrate was selected for bioethanol production by *B. subtilis* AP. This bacterium was an engineered ethanologenic strain that was evaluated in this study for the valorization of wheat bran as a crop waste. As shown in Fig. 5a, the ethanol concentration in PBX medium (4.52 g/L) was 1.8 and 2.4 folds higher than PB and UB mediums after 48 h, respectively. During the next 48 h of fermentation, the ethanol concentration in PB and UB mediums raised to about 5.5 g/L while in PBX medium was 7.33 g/L. The results suggest that *B. subtilis* AP by its capacity to decompose wheat bran could gradually compensate for the lack of the mild substrate pretreatment during 96 h of fermentation, producing almost the same amount of ethanol in UB medium as was produced in PB medium. In addition, this strain could recompense significantly the lack of external Xyn-2 addition and produced a rather high titer of ethanol in UB and PB mediums as compared to the PBX medium. The measurement of the residual biomass from UB, PB, and PBX mediums at the end of fermentation revealed that about 64.4%, 64.9%, and 73% of the initial solid biomass has been dissolved into the culture mediums, respectively (Fig. 5b). While there was no significant difference in the efficiency of wheat bran utilization between UB and PB cultures, the efficiency was significantly higher for PBX culture which may be attributed to the function of Xyn-2. The results confirmed that *B. subtilis* AP is able to produce ethanol from wheat bran in both CBP and SSF systems. The ethanol production yields were calculated as being 22.6% and 26.8% of the theoretical value, respectively. *B. subtilis* is known as a bacterium with a remarkable ability to utilize various carbon sources due to the production and secretion of a variety of enzymes^33^. These enzymes enable *B. subtilis* AP to grow and produce ethanol on wheat bran in CBP. However, the significant contribution of Xyn-2 to ethanol production by *B. subtilis* AP in PBX medium suggests that the heterologous expression of select enzymes would be of a great impact on the CBP ethanol production by this bacterium.

## Discussion

Metagenomes are an invaluable source of novel genes that may not be obtained otherwise. The naturally evolved genes may be expressed in a surrogate host and screened for a desired function or activity. The gene *xyn-2* of the current study was isolated from a camel rumen metagenome and identified as coding for a novel GH10 family psychrohalophilic xylanase. The metagene showed no identity to other genes deposited at NCBI except very low identity to a gene from *Prevotella ruminicola*.

The cold-adapted enzymes usually have an optimum activity temperature of about 20–30 °C^34^. Interestingly, Xyn-2 with an optimum temperature of 20 °C and keeping 58% of its activity at 0 °C might be assumed as the most psychrophilic xylanase reported so far. Xyn-2 is also outstanding for exhibiting two times elevated activity in the presence of high NaCl concentrations. Table 1 contains characterized xylanases that are either psychrophilic or halophilic or both. Although several hundred xylanases have been studied so far, just a few have been cold-adapted or halophilic, among which Xyn-2 is the most cold-adapted halophilic xylanase while its activity is doubled in a wide range of high salt concentrations (0.5–4 M). To have a better comparison between Xyn-2 and other enzymes listed in Table 1, the salt concentration required for the highest activity of each enzyme, and the amount of salt-induced activities are also included (Table 1).

**Table 1:** Comparison of optimum temperature, pH, and NaCl concentration between Xyn-2 and other cold-adapted and/or halophilic xylanases.

Xylanase (GH family)	Source	Opt. Temp. (°C)	Activity at 0 °C (%)	Opt. pH	Salt Con. for Max. activity (M)	Max activity induction (folds)	Ref
Xyn-2 (GH10)	Camel rumen metagenome	20	58	5	0.5–4	2	This study
XylCMS (GH11)	Camel rumen metagenome	55	0	6	3	1.4	^36^
XynA (GH10)	*Thermoanaerobacterium saccharolyticum* NTOU1	72	14	5.5	0.4	1.9	^37^
LaXynA (GH10)	*Luteimonas abyssi* XH031T	30	29	6.5	0.5	4	^38^
Xyn10C (10)	*Saccharophagus degradans* 2–40	30	–	7	1.5–2	1.9	^39^
Xyn11 (GH11)	*Bispora antennata*	35	21	5.5	–	–	^40^
XynA (GH10)	*Zunongwangia profunda*	30	23	6.5	1.5–3	1.8	^41^
Xyn10A (GH10)	*Flavobacterium johnsoniae*	30	50	8	–	–	^42^
XynA (GH10)	*Sorangium cellulosum* So9733-1	30	13.7	7	–	–	^43^
XynA (GH10)	*Glaciecola mesophila* KMM 241	30	12	7	0.5–1	1.2	^44^

*GH family* glycoside hydrolase family, *Opt.* optimum, *Temp.* temperature, *Con.* Concentration, *M* molarity, *Max* maximum.

Psychrophilic xylanases like Xyn-2 are promising to find application in various industries where reactions are conducted at moderate temperatures. Cold-active enzymes are industrially important due to their ability to function well at ambient temperature reducing energy consumption by eliminating the need for heating^35^. In food industries, such an enzyme might be used in dough processing to make more tender and less staling breads^45^. In this regard, a psychrophilic xylanase from the Antarctic bacterium *P. haloplanktis* has been commercialized by Puratos (Belgium)^46^. Moreover, an interesting area for the application of halophilic xylanases would be in biorefineries to replace freshwater consumption with abundant seawater. Given the resource shortages of fresh water in many parts of the world, the use of seawater in industries seems reasonable or even inevitable in the future. In addition, inorganic salt has successfully been used as an efficient pretreatment for the liberation of reducing sugars from plant biomass^47^. Salt pretreatment results in costs reduction compared with other pretreatment methods, and it might give rise to the higher efficiency of other pretreatment methods in integrated technologies^48^. In this regard, one important feature that distinguishes Xyn-2 from other halophilic xylanases is the ability of the enzyme to exhibit its maximum salt-induced activity over a wide range of NaCl concentrations from 0.5 M (as in seawater) to 4 M (Table 1).

In addition, several groups of chemicals including reducing and oxidizing agents, ions, EDTA, and denaturants were used to study their effects on the enzyme activity. The resulting data help to gain an insight into the unique features of Xyn-2 and the reaction conditions that lead to the maximum achievable activity of the enzyme. Some enzymes are strictly dependent on specific metal ions to remain active. The activity of other enzymes might also be significantly elevated or inhibited by some ions^49^. Xyn-2 proved in this study to be neither a metalloenzyme nor an enzyme that its activity is severely inhibited by the cations. This is a significant feature of an enzyme for industrial application where ions are highly likely to enter the reaction mixture due to the necessity of using inexpensive feedstocks instead of analytical-grade ones.

Likewise, the ability of Xyn-2 to tolerate and even exhibit induced activities in high concentrations of urea is remarkable. Urea has been suggested as an inexpensive chemical for efficient pretreatment of plant biomass before enzymatic saccharification^50,51^. Therefore, Xyn-2 may be a suitable partner to urea in the development of new technologies for biofuel production or animal feed preparation^52^.

In this study, Xyn-2 was evaluated for saccharification of wheat bran for bioethanol production. For this purpose, the ethanologenic *B. subtilis* AP was used to conduct fermentation. *B. subtilis* is well known for its ability to survive on a variety of substrates. The safety of the bacterium as a GRAS (generally regarded as safe) microorganism as well as its sophisticated systems for production and secretion of enzymes have encouraged the development of ethanologenic *B. subtilis* AP^23^. This study proved the potential of *B. subtilis* AP for bioethanol production from wheat bran. Although a mild pretreatment by the artificial seawater plus HCl could have an early positive impact on ethanol production, it had no significant impact on the final ethanol concentration compared to untreated biomass. It may be assumed that under the experimental conditions of this study the impact of the mild chemical treatment could be compensated by the inherent enzymatic potentials of *B. subtilis*. This observation might be taken as proof of the merits of *B. subtilis* for bioconversion of plant biomass. Table 2 provides a comparison between different studies conducted on ethanol production from wheat bran.

**Table 2:** Bioethanol production by various organisms using raw wheat bran (RWB) and milled wheat bran (MWB) in different studies.

Substrate	Pretreatment	Enzymatic hydrolysis	Strain	Process	EtOH (g/L )	Reference
RWB	–	–	*B. subtilis* AP	CBP	5.5	This study
RWB	120 °C, 20 min 0.3% HCl in seawater	Xyn-2	*B. subtilis* AP	SSF	7.3	This study
RWB	–	–	*Trametes hirsute*	CBP	4.3	^54^
RWB	190 °C, 10 min phosphoric acid (1.75%)	Cellic Ctec2	*Neurospora intermedia*	SHF	9	^55^
RWB and MWB	121 °C, 20 min H2SO4 (3%)	Novozymes Biomass Kit and Liquozyme	*S. cerevisiae* s1 or *S. diastaticus*	SHF	10	^56^
RWB	121 °C, 25 min H2SO4 (1%)	Novozymes Biomass Kit	*S. cerevisiae* (DSM70449) and *S. cerevisiae* MEL2	SHF	12	^57^
MWB	121 °C, 60 min	α-amylase (Diastase)	*A. niger* (MTCC 1349) and *K. marxianus* (MTCC1389)	SHF and SSF	23.1	^53^
RWB	121 °C, 15 min	–	*Cotylidia pannosa* and *Saccharomyces cerevisiae* (MTCC 174)	SHF	4.12	^58^

*CBP* consolidated bioprocessing, *SSF* simultaneous saccharification and fermentation, *SHF* separate hydrolysis and fermentation.

In contrast to the present study, other studies previously conducted on bioethanol production from wheat bran mostly had used more severe physicochemical pretreatments along with highly efficient commercial enzymes. Only Okamoto et al. used the CBP system for bioethanol production from wheat bran in a similar way to the present study. *Trametes hirsute* used by Okamoto et al. is a naturally ethanologenic fungus that can efficiently hydrolyze cellulose, hemicellulose, and lignin by its lignocellulolytic enzymes^54^. Compared to *Trametes hirsute, B. subtilis* AP as an engineered strain seems quite interesting in its potential for CBP ethanol production from wheat bran. CBP ethanol production is industrially more favored than SSF system due to its potential to reduce costs by removing the consumption of commercial enzymes^59^.

## Conclusion

The saccharification of plant biomass to fermentable sugars is a major bottleneck in fermentation technology because of the recalcitrance of plant lignocellulosic structure. Although harsh chemical and intensive enzymatic treatments can be effective, the environmental impacts, high expenses, and formation of toxic compounds are the main drawbacks to saccharification and fermentation of lignocellulosic biomass. The metagenomic xylanase Xyn-2 obtained from camel rumen in this study proved to be highly efficient in the saccharification of plant biomass wastes. The results indicated that the camel rumen metagenome is a valuable source of beneficial xylanases and possibly other cellulolytic enzymes. In this study, the ethanologenic *B. subtilis* AP was successfully used for ethanol production from wheat bran in CBP and SSF processes. The strain as an engineered ethanologenic prototype proved to be quite comparable with naturally ethanologenic microorganisms that have already been used for this purpose. The significant contribution of Xyn-2 to ethanol production of this strain in SSF process suggests that heterologous expression of efficient cellulolytic enzymes in *B. subtilis* AP would lead to a more potent strain for CBP ethanol production from plant biomass.

## Supplementary Information

Supplementary Information 1.

Supplementary Information 2.

Supplementary Figure S1.

Supplementary Figure S2.

Supplementary Figure S3.

## References

[1] Gírio FM (2010). Hemicelluloses for fuel ethanol: A review. Biores. Technol..

[2] Di Gruttola F, Borello D (2021). Analysis of the EU secondary biomass availability and conversion processes to produce advanced biofuels: Use of existing databases for assessing a metric evaluation for the 2025 Perspective. Sustainability.

[3] Raj T (2022). Recent advances in commercial biorefineries for lignocellulosic ethanol production: Current status, challenges and future perspectives. Biores. Technol..

[4] Sims RE, Mabee W, Saddler JN, Taylor M (2010). An overview of second generation biofuel technologies. Biores. Technol..

[5] Brown LM, Hawkins GM, Doran-Peterson J (2017). Ethanol production from renewable lignocellulosic biomass. Biofuels Bioenergy.

[6] Halder, P., Azad, K., Shah, S. & Sarker, E. in *Advances in Eco-Fuels for a Sustainable Environment* 211–236 (Elsevier, 2019).

[7] Collins T, Gerday C, Feller G (2005). Xylanases, xylanase families and extremophilic xylanases. FEMS Microbiol. Rev..

[8] Thomas T, Gilbert J, Meyer F (2012). Metagenomics-a guide from sampling to data analysis. Microb. Inf. Exp..

[9] Terrapon, N., Lombard, V., Drula, E., Coutinho, P. M. & Henrissat, B. in *A Practical Guide to Using Glycomics Databases* 117–131 (Springer, 2017).

[10] Bhardwaj N, Kumar B, Verma P (2019). A detailed overview of xylanases: an emerging biomolecule for current and future prospective. Bioresour. Bioprocess..

[11] Burlacu A, Cornea CP, Israel-Roming F (2016). Microbial xylanase: A review. Sci. Bull. Ser. F. Biotechnol..

[12] Kumar L, Nagar S, Mittal A, Garg N, Gupta VK (2014). Immobilization of xylanase purified from *Bacillus pumilus* VLK-1 and its application in enrichment of orange and grape juices. J. Food Sci. Technol..

[13] Wu H (2021). Multimodularity of a GH10 xylanase found in the termite gut metagenome. Appl. Environ. Microbiol..

[14] Ellilä S (2019). Cloning of novel bacterial xylanases from lignocellulose-enriched compost metagenomic libraries. AMB Express.

[15] Knapik K, Becerra M, González-Siso M-I (2019). Microbial diversity analysis and screening for novel xylanase enzymes from the sediment of the Lobios Hot Spring in Spain. Sci. Rep..

[16] Kim H-B, Lee K-T, Kim M-J, Lee J-S, Kim K-S (2018). Identification and characterization of a novel KG42 xylanase (GH10 family) isolated from the black goat rumen-derived metagenomic library. Carbohyd. Res..

[17] Rashamuse K (2013). Characterisation of two bifunctional cellulase–xylanase enzymes isolated from a bovine rumen metagenome library. Curr. Microbiol..

[18] Ghadikolaei KK, Noghabi KA, Zahiri HS (2017). Development of a bifunctional xylanase-cellulase chimera with enhanced activity on rice and barley straws using a modular xylanase and an endoglucanase procured from camel rumen metagenome. Appl. Microbiol. Biotechnol..

[19] Feller G (2010). Protein stability and enzyme activity at extreme biological temperatures. J. Phys. Condens. Matter.

[20] Mangiagalli M, Lotti M (2021). Cold-active β-galactosidases: Insight into cold adaptation mechanisms and biotechnological exploitation. Mar. Drugs.

[21] Maes C, Delcour J (2001). Alkaline hydrogen peroxide extraction of wheat bran non-starch polysaccharides. J. Cereal Sci..

[22] Green Michael R, Sambrook J (2012). Molecular cloning: A Laboratory Manual.

[23] Maleki F (2021). Consolidated bioprocessing for bioethanol production by metabolically engineered *Bacillus subtilis* strains. Sci. Rep..

[24] Gharechahi J, Zahiri HS, Noghabi KA, Salekdeh GH (2015). In-depth diversity analysis of the bacterial community resident in the camel rumen. Syst. Appl. Microbiol..

[25] Peng Y, Leung HC, Yiu S-M, Chin FY (2012). IDBA-UD: a de novo assembler for single-cell and metagenomic sequencing data with highly uneven depth. Bioinformatics.

[26] Bankevich A (2012). SPAdes: a new genome assembly algorithm and its applications to single-cell sequencing. J. Comput. Biol..

[27] Hyatt D, LoCascio PF, Hauser LJ, Uberbacher EC (2012). Gene and translation initiation site prediction in metagenomic sequences. Bioinformatics.

[28] Galperin MY, Kristensen DM, Makarova KS, Wolf YI, Koonin EV (2019). Microbial genome analysis: The COG approach. Brief. Bioinform..

[29] Mistry J (2021). Pfam: The protein families database in 2021. Nucleic Acids Res..

[30] Overbeek R (2014). The SEED and the Rapid Annotation of microbial genomes using Subsystems Technology (RAST). Nucleic Acids Res..

[31] Kanehisa M, Furumichi M, Sato Y, Ishiguro-Watanabe M, Tanabe M (2021). KEGG: Integrating viruses and cellular organisms. Nucleic Acids Res..

[32] Bose T, Haque MM, Reddy C, Mande SS (2015). COGNIZER: A framework for functional annotation of metagenomic datasets. PLoS ONE.

[33] Westers L, Westers H, Quax WJ (2004). *Bacillus subtilis* as cell factory for pharmaceutical proteins: a biotechnological approach to optimize the host organism. Biochimica et Biophysica Acta Mol. Cell Res..

[34] Mangiagalli M, Brocca S, Orlando M, Lotti M (2020). The “cold revolution”. Present and future applications of cold-active enzymes and ice-binding proteins. New Biotechnol..

[35] Santiago M, Ramírez-Sarmiento CA, Zamora RA, Parra LP (2016). Discovery, molecular mechanisms, and industrial applications of cold-active enzymes. Front. Microbiol..

[36] Ghadikolaei KK, Sangachini ED, Vahdatirad V, Noghabi KA, Zahiri HS (2019). An extreme halophilic xylanase from camel rumen metagenome with elevated catalytic activity in high salt concentrations. AMB Express.

[37] Hung K-S (2011). Characterization of a salt-tolerant xylanase from *Thermoanaerobacterium saccharolyticum* NTOU1. Biotech. Lett..

[38] Han Z, Shang-Guan F, Yang J (2018). Characterization of a novel cold-active xylanase from *Luteimonas* species. World J. Microbiol. Biotechnol..

[39] Ko JK, Ko H, Kim KH, Choi I-G (2016). Characterization of the biochemical properties of recombinant Xyn10C from a marine bacterium, *Saccharophagus degradans* 2–40. Bioprocess. Biosyst. Eng..

[40] Liu Q (2015). Isolation of a novel cold-active family 11 xylanase from the filamentous fungus *Bispora antennata* and deletion of its N-terminal amino acids on thermostability. Appl. Biochem. Biotechnol..

[41] Liu X, Huang Z, Zhang X, Shao Z, Liu Z (2014). Cloning, expression and characterization of a novel cold-active and halophilic xylanase from *Zunongwangia profunda*. Extremophiles.

[42] Chen S, Kaufman MG, Miazgowicz KL, Bagdasarian M, Walker ED (2013). Molecular characterization of a cold-active recombinant xylanase from *Flavobacterium johnsoniae* and its applicability in xylan hydrolysis. Biores. Technol..

[43] Wang S-Y, Hu W, Lin X-Y, Wu Z-H, Li Y-Z (2012). A novel cold-active xylanase from the cellulolytic *Myxobacterium Sorangium* cellulosum So9733-1: Gene cloning, expression, and enzymatic characterization. Appl. Microbiol. Biotechnol..

[44] Guo B, Chen X-L, Sun C-Y, Zhou B-C, Zhang Y-Z (2009). Gene cloning, expression and characterization of a new cold-active and salt-tolerant endo-β-1, 4-xylanase from marine *Glaciecola mesophila* KMM 241. Appl. Microbiol. Biotechnol..

[45] Collins T (2002). A novel family 8 xylanase, functional and physicochemical characterization. J. Biol. Chem..

[46] Bhatia RK (2021). Psychrophiles: A source of cold-adapted enzymes for energy efficient biotechnological industrial processes. J. Environ. Chem. Eng..

[47] Loow Y-L (2015). Recent advances in the application of inorganic salt pretreatment for transforming lignocellulosic biomass into reducing sugars. J. Agric. Food Chem..

[48] Loow Y-L (2018). Deep eutectic solvent and inorganic salt pretreatment of lignocellulosic biomass for improving xylose recovery. Biores. Technol..

[49] Ding C, Li M, Hu Y (2018). High-activity production of xylanase by *Pichia stipitis*: Purification, characterization, kinetic evaluation and xylooligosaccharides production. Int. J. Biol. Macromol..

[50] Wang L, Zhang K, Xu Y, Zhang M, Wang D (2018). High-solid pretreatment of corn stover using urea for enzymatic saccharification. Biores. Technol..

[51] Wang Q, Wei W, Kingori GP, Sun J (2015). Cell wall disruption in low temperature NaOH/urea solution and its potential application in lignocellulose pretreatment. Cellulose.

[52] Xu Y (2019). Effects of incremental urea supplementation on rumen fermentation, nutrient digestion, plasma metabolites, and growth performance in fattening lambs. Animals.

[53] Manikandan K, Viruthagiri T (2009). Simultaneous saccharification and fermentation of wheat bran flour into ethanol using coculture of amylotic *Aspergillus niger* and thermotolerant *Kluyveromyces marxianus*. Front. Chem. Eng. China.

[54] Okamoto K, Nitta Y, Maekawa N, Yanase H (2011). Direct ethanol production from starch, wheat bran and rice straw by the white rot fungus *Trametes hirsuta*. Enzyme Microb. Technol..

[55] Nair RB, Lundin M, Brandberg T, Lennartsson PR, Taherzadeh MJ (2015). Dilute phosphoric acid pretreatment of wheat bran for enzymatic hydrolysis and subsequent ethanol production by edible fungi *Neurospora intermedia*. Ind. Crops Prod..

[56] Favaro L, Basaglia M, Casella S (2012). Processing wheat bran into ethanol using mild treatments and highly fermentative yeasts. Biomass Bioenerg..

[57] Favaro L, Basaglia M, van Zyl WH, Casella S (2013). Using an efficient fermenting yeast enhances ethanol production from unfiltered wheat bran hydrolysates. Appl. Energy.

[58] Sharma D (2018). Endocellulase production by *Cotylidia pannosa* and its application in saccharification of wheat bran to bioethanol. Bioenergy Res..

[59] Su T, Zhao D, Khodadadi M, Len C (2020). Lignocellulosic biomass for bioethanol: Recent advances, technology trends and barriers to industrial development. Curr. Opin. Green Sustain. Chem..

