# Supplementary material for: Metagenomic psychrohalophilic xylanase from camel rumen investigated for bioethanol production from wheat bran using Bacillus subtilis AP
Source: Sci Rep. 2022 May 17;12:8152. doi: 10.1038/s41598-022-11412-4 (PMC9114127; doi:10.1038/s41598-022-11412-4)
Supplement: Supplementary file 3 — Supplementary Figure S1. [file 41598_2022_11412_MOESM3_ESM.pdf]

Supplementary figure S1

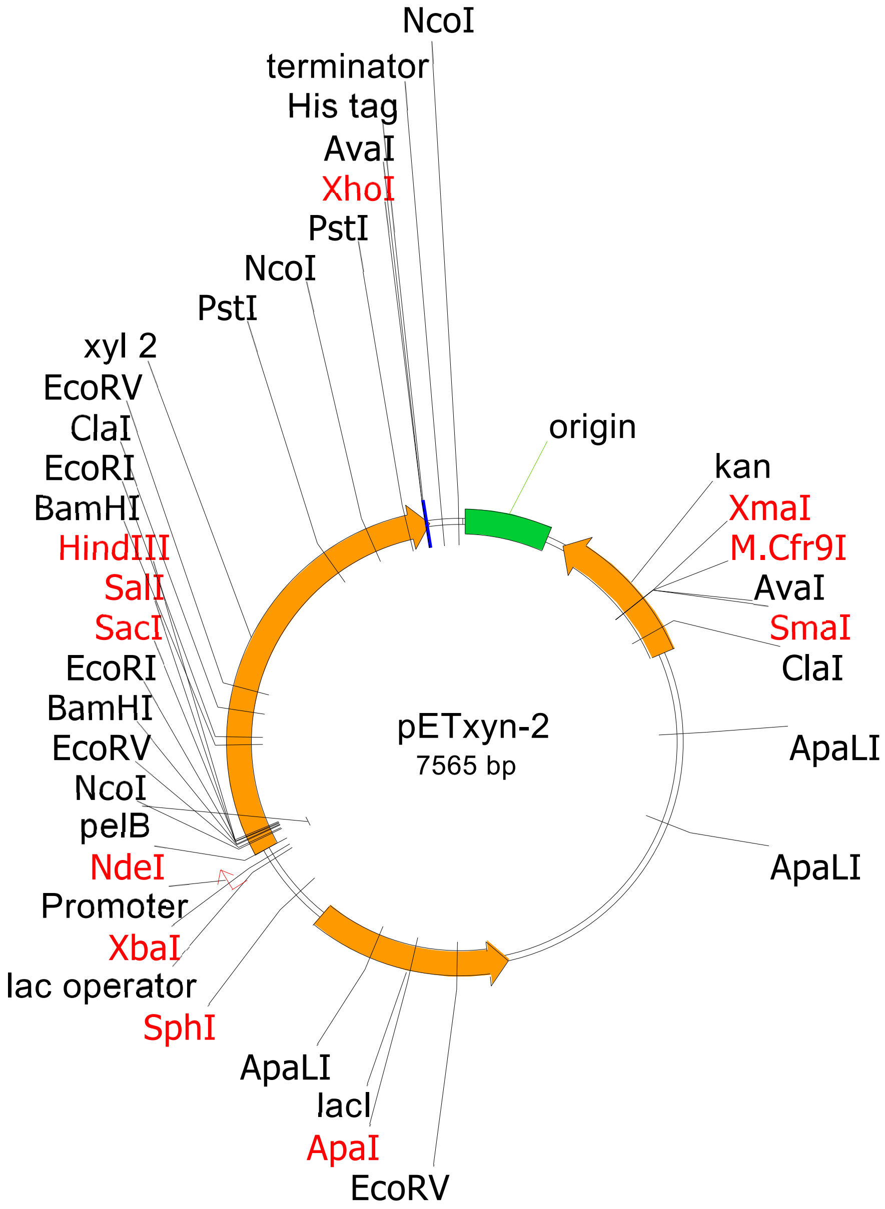

Figure S1. Plasmid map of pETxyn-2. This plasmid was constructed by inserting the gene encoding Xyn-2 into pET-26b(+).
