# Supplementary material for: Metagenomic psychrohalophilic xylanase from camel rumen investigated for bioethanol production from wheat bran using Bacillus subtilis AP
Source: Sci Rep. 2022 May 17;12:8152. doi: 10.1038/s41598-022-11412-4 (PMC9114127; doi:10.1038/s41598-022-11412-4)
Supplement: Supplementary file 4 — Supplementary Figure S2. [file 41598_2022_11412_MOESM4_ESM.pdf]

Supplementary figure S2

```
                    1                                                50
FJ713437.1     (1)  ----MKKLLVALSLIAGSLTASAQWGRPVDYAAGPGLKDAYKDYFTVGVA
KX644148.1     (1)  MKQSFKKALVVALVMGGTTSVWAQG-----------LKDAYKDYFRIGVA
                    51                                              100
FJ713437.1    (47)  VNKFNISDPAQTAIVKKQFNSVTAENAWKPGEIHPKEGVWNFGLADSIAN
KX644148.1    (40)  LNQRNIADAEQQALVKREFNSITAENDMKPQLTEPNEGEFTWEAADRIAN
                    101                                             150
FJ713437.1    (97)  FCRENGIKMRGHCLCWHSQFADWMFTDKKGKPVKKEVFYQRLREHIHTVV
KX644148.1    (90)  FCRQNGIKLRGHCLMWHSQIGEWMYKDN----PTKEVLFARMRKHIHAVV
                    151                                             200
FJ713437.1   (147)  NRYKDVVYAWDVVNEAMADDGRPFEFVDGKMVPASPYRQSRHFKLCGDEF
KX644148.1   (136)  SRYKDVVYCWDVVNEAMTDDKN----------AMDPYRQSQFYKIAGDEF
                    201                                             250
FJ713437.1   (197)  IAKAFEFAREADPTGVLMYNDYSCVDEGKRERIYNMVKKMKEAGVPIDGI
KX644148.1   (176)  IAKAFQYAREADPKALLFYNDYNECDPVKSQRIYNMVKAMKQAGVPIDGI
                    251                                             300
FJ713437.1   (247)  GMQGHYNIYFPDEEKLEKAINRFSEIVNTIHITELDLRTNTESGGQLMFS
KX644148.1   (226)  GMQGHYNIYGPTEKEVDDAITLYKKIVKHIHVTELDIRVNTEMGGQLRFS
                    301                                             350
FJ713437.1   (297)  RGEAKPQPGYMQTLQEDQYARLFKIFRKHKDVIKNVTFWNLSDKDSWLGV
KX644148.1   (276)  RG-GVTVSDSIKQHLADQYARVFKVFRKHKDVIDCVTFWNLGDRDSWLGA
                    351                                             400
FJ713437.1   (347)  NNHPLPFDENFKAKRSLQIIRDFDAAMDNR------KP------------
KX644148.1   (325)  ANYPLPFDSEYKPKLAYDFIKNMKPAKWELPTAPAPKPRQQQMERPQFNA
                    401                                             450
FJ713437.1   (379)  ----------KEDFVPNPMNQPGQEYPMVNSEGYARFRVEAPDAKSVIVS
KX644148.1   (375)  RMAFRPIEGVKDDFKPSEMNQPGQEYPMVNSQGYVRFRVMAPDAKAVSVS
                    451                                             500
FJ713437.1   (419)  LGLGGRGGTVLRKDKNGVWTGTTEGPMDPGFHYYHLTIDGGVFNDPGTHN
KX644148.1   (425)  LGLGGQGGTKLRRFFDGSWIGTTEGPMDEGFHYYHLTVDGGTLNDPGTQN
                    501                                             550
FJ713437.1   (469)  YFGSCRWESGIEIPAKDQDFYAYRKDINHGNIQQVTFWSESTGKMQTANV
KX644148.1   (475)  YFGSCRWESGIEIPAHDADFYAMKN-VPHGNVQQVLFWSESTKQVRRAMV
                    551                                             600
FJ713437.1   (519)  YLPYGYGKVVKGKQERYPVLYLQHGWGENETSWPVQGKAGLIMDNLIADG
KX644148.1   (524)  YTPPTYGQNKK---EKYPVLYLQHGWGEDETAWSRQGHANLIMDNLIAEG
                    601                                             650
FJ713437.1   (569)  KIKPFIVVMAYGLTNDFKFGSIGKFTAEEFEKVLIDELIPTIDKNFLTKA
KX644148.1   (571)  KIQPFIVVMTYGMTNDVKFGTIGQFTAKEFETVLVDELVPYIDSHFQTKA
                    651                                             700
FJ713437.1   (619)  DKWNRAMAGLSMGGMETKLITLRRPEMFGYWGLLSGGTYMPEEIKDPKAV
KX644148.1   (621)  DKWNRAMAGLSMGGVETKLITLRRPETFGYYGLLSGGMYAPEDIKDKSQV
                    701                                             750
FJ713437.1   (669)  KYIFVGCGDKENPEGINKSVEALKAAGFKAEGLVSEGTAHEFLTWRRCLE
KX644148.1   (671)  RMIFQSCGSKENPDGIRKSVEALKAAGFNAHGYVSEGTAHEFLTWRRSLK
                    751
FJ713437.1   (719)  KMAQSLFK-
KX644148.1   (721)  EMAPLLFKK
```

Figure S2. Alignment of translated sequence of *xyn-2* (GenBank: KX644148.1) with its closest relative from *Prevotella ruminicola* (GenBank: FJ713437.1).
