# Supplementary material for: Metagenomic psychrohalophilic xylanase from camel rumen investigated for bioethanol production from wheat bran using Bacillus subtilis AP
Source: Sci Rep. 2022 May 17;12:8152. doi: 10.1038/s41598-022-11412-4 (PMC9114127; doi:10.1038/s41598-022-11412-4)
Supplement: Supplementary file 5 — Supplementary Figure S3. [file 41598_2022_11412_MOESM5_ESM.pdf]

Supplementary figure S3

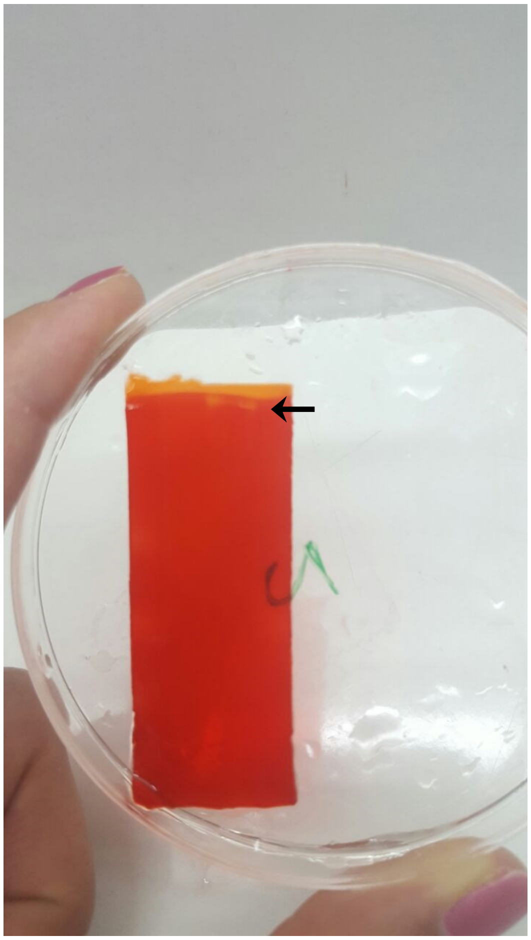

Figure S4. Native PAGE and activity staining of Xyn-2 with Congo-red. The arrow indicates the yellowish band of xylan hydrolysis.
